# Cross‐Ray Ultrasound Tomography and Photoacoustic Tomography of Cerebral Hemodynamics in Rodents

**DOI:** 10.1002/advs.202201104

**Published:** 2022-07-11

**Authors:** Shuai Na, Yang Zhang, Lihong V. Wang

**Affiliations:** ^1^ Caltech Optical Imaging Laboratory Andrew and Peggy Cherng Department of Medical Engineering California Institute of Technology Pasadena CA 91125 USA; ^2^ Department of Electrical Engineering California Institute of Technology Pasadena CA 91125 USA; ^3^ Present address: National Biomedical Imaging Center, College of Future Technology Peking University Beijing 100871 China

**Keywords:** brain imaging, cross‐ray ultrasound tomography, Doppler imaging, photoacoustic tomography

## Abstract

Recent advances in functional ultrasound imaging (fUS) and photoacoustic tomography (PAT) offer powerful tools for studying brain function. Complementing each other, fUS and PAT, respectively, measure the cerebral blood flow (CBF) and hemoglobin concentrations, allowing synergistic characterization of cerebral hemodynamics. Here, cross‐ray ultrasound tomography (CRUST) and its combination with PAT are presented. CRUST employs a virtual point source from a spherically focused ultrasonic transducer (SFUST) to provide widefield excitation at a 4‐kHz pulse repetition frequency. A full‐ring‐shaped ultrasonic transducer array whose imaging plane is orthogonal to the SFUST's acoustic axis receives scattered ultrasonic waves. Superior to conventional fUS, whose sensitivity to blood flow is angle‐dependent and low for perpendicular flow, the crossed transmission and panoramic detection fields of CRUST provide omnidirectional sensitivity to CBF. Using CRUST‐PAT, the CBF, oxygen saturation, and hemoglobin concentration changes of the mouse brain during sensory stimulation are measured, with a field of view of ≈7 mm in diameter, spatial resolution of ≈170 µm, and temporal resolution of 200 Hz. The results demonstrate CRUST‐PAT as a unique tool for studying cerebral hemodynamics.

## Graphical abstract

Cross‐ray ultrasound tomography (CRUST) consists of a standalone spherically focused ultrasonic transducer for transmission and an orthogonal ring‐shaped array for detection. The crossed transmission and panoramic detection fields provide omnidirectional Doppler sensitivity, distinguishing CRUST from conventional methods. CRUST and photoacoustic tomography are combined to image the dynamics of cerebral blood flow and hemoglobin concentrations simultaneously in the mouse brain.

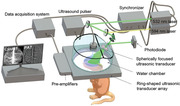

## 1 Introduction

Cerebral blood flow (CBF) provides the key to unraveling neurovascular coupling in response to neuronal activities.^[^ ^1^ ^]^ Label‐free optical and Doppler ultrasound methods have been widely employed to image CBF.^[^ ^2^ ^]^ The optical methods fall into three main categories: Doppler‐based laser flowmetry, red blood cell (RBC)‐tracking‐based laser scanning microscopy, and speckle‐based imaging.^[^ ^3^, ^4^, ^5^ ^]^ The first two categories employ point scanning and offer quantitative measurements at the expense of the field of view (FOV). Speckle‐based methods, for example, laser speckle contrast imaging and multi‐exposure speckle imaging, provide high spatiotemporal resolution with a large FOV, but they require mathematical modeling and calibration for quantitative flow measurements.^[^ ^6^ ^]^ Additionally, these optical methods have limited imaging depths. Due to its low cost, flexibility, and ability to provide quantitative measurements at a greater depth, Doppler ultrasound has been extensively used for preclinical and clinical neuroimaging.^[^ ^7^ ^]^ Conventional Doppler ultrasound imaging scans the region of interest using a focused beam line by line at a frame rate (FR) typically below 60 Hz (corresponding to 5 cm in depth and 256 lines in width). This scanning method limits the FOV and produces aliasing artifacts in measurements of high‐velocity blood flow. Ultrafast ultrasound imaging capitalizes on successive tilted plane wave transmissions, which are coherently compounded to form images at a few hundred to several thousand hertz, enabling functional ultrasound imaging (fUS).^[^ ^8^, ^9^ ^]^ Despite its advantages, fUS inherently lacks molecular specificity and suffers decreased sensitivity to blood flowing perpendicularly to the acoustic axis. For brain imaging, the standard protocol requires the ultrasonic transducer probe to be in direct contact with the head, so blood vessels distributed along the cortical surface are not well resolved.^[^ ^10^ ^]^

Complementing fUS with molecular specificity, photoacoustic (PA) tomography (PAT) uses thermo‐elastically induced acoustic waves to measure the optical absorption of endogenous hemoglobin (Hb).^[^ ^11^ ^]^ PAT's widely used panoramic detection scheme provides high sensitivity to blood vessels along the cortical surface over a large FOV.^[^ ^12^ ^]^ Also, images of acoustically smooth blood vessel boundaries suppress interior speckles.^[^ ^13^ ^]^ In PAT, full‐ or wide‐angular‐view ultrasonic transducer arrays (USTAs) provide better imaging quality than linear or planar arrays.^[^ ^14^, ^15^, ^16^, ^17^ ^]^ To suppress the limited‐view problem, USTAs may adopt 2D ring‐shaped or 3D bowl‐shaped configurations tailored for maximum 2D and 3D detection coverages, respectively.^[^ ^18^ ^]^ Nevertheless, the relatively large element size and pitch of these USTAs hamper the implementation of fUS: the highly diffractive sidelobes prevent proper beam steering.^[^ ^19^, ^20^ ^]^ Moreover, equipping a PAT system with fUS capability requires considerable hardware modification. For example, it is common to add pulsers for digital beamforming and switches for rapid switching between the transmission and receiving events, and these electronics generally need to be one‐to‐one‐mapped to the transducer elements to achieve a sufficiently high FR for fUS.^[^ ^19^, ^20^, ^21^, ^22^ ^]^ Overall, achieving vector flow imaging, especially with omnidirectional sensitivity and minimal hardware modification of an established PAT system, remains challenging.

In this work, we propose cross‐ray ultrasound tomography (CRUST), which provides omnidirectional sensitivity to blood flow and an easy‐to‐implement solution to merging fUS and PAT. CRUST employs a virtual point acoustic source from a standalone spherically focused ultrasonic transducer (SFUST) to transmit spherical waves to the far field at an ultrahigh pulse repetition frequency (PRF). It shares the detection aperture of an established PAT system with a 512‐element elevationally focused full‐ring‐shaped USTA. Scattered acoustic waves, such as Rayleigh‐scattered waves from RBCs in the imaging plane are detected. CRUST images are reconstructed by extending the inverse spherical Radon transform to the ellipsoidal form.^[^ ^23^ ^]^ Unlike conventional fUS, the transmitted and scattered ultrasound rays in CRUST are crossed, allowing for angle‐independent flow measurements.^[^ ^24^ ^]^ Here, we validate CRUST through phantom studies by imaging blood‐mimic fluid (BMF) at various controlled flow velocities in microtubes. By imaging mouse brain functioning during hindlimb electrical stimulation, we demonstrate CRUST‐PAT as a unique tool providing cerebral vascular morphology, CBF mapping, hemoglobin concentration, and oxygenation measurements simultaneously, all with a large FOV and high spatiotemporal resolution.

## 2 Methods

### 2.1 CRUST‐PAT Implementation

**Figure 1:**
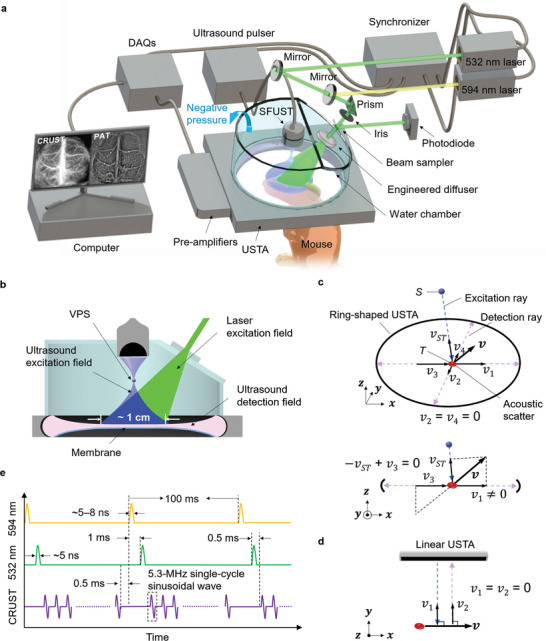
CRUST‐PAT implementation. a) Perspective view of the CRUST‐PAT system. b) Cut‐away view showing the ultrasound excitation, PA excitation, and acoustic detection fields. c) Perspective view (top) and side view (bottom) of CRUST for flow imaging. *S*, the position of the VPS; *T*, the position of the acoustic scatterer; *v*, blood flow velocity in the *xz* plane; *v_ST_*, velocity projection coefficient along the excitation ray; *v*_1_–*v*_4_, velocity projection coefficients along the detection rays. Even for flow velocity with equal projection coefficients along the excitation and detection rays (i.e., − *v_ST_* + *v*_3_ = 0), there always exist nonzero projection coefficients along other detection rays (e.g., *v*_1_ ≠ 0). d) Conventional Doppler ultrasound imaging of the blood flow. e) Excitation sequences of CRUST and PAT.

As illustrated in **Figure** **1**a, CRUST‐PAT consists of an SFUST for ultrafast ultrasound excitation in the far field, a dual‐wavelength laser system for PA excitation, and a full‐ring‐shaped USTA for ultrasound detection. Driven by an electrical pulser consisting of a waveform generator (DG4162, Rigol, Inc.) and a broadband 50‐dB power amplifier (Model 240L, ENI, Inc.), the SFUST (5.3‐MHz central frequency, numerical aperture of 0.5, ≈78% one‐way fractional bandwidth at −3 dB, A307‐SU, Olympus, Corp.) can fire ultrasound pulses at a tunable PRF. The USTA, designed in‐house and custom‐built by Imasonic, Inc., consists of 512 elements centered at 5.4 MHz, with a ≈80% one‐way fractional bandwidth of −3 dB. It has a 10‐cm diameter and a 2‐cm elevational element size, with a 0.2 numerical aperture, a 0.61‐mm pitch, and a 0.1‐mm kerf.^[^ ^14^ ^]^ The SFUST is positioned with its acoustic axis orthogonal to the USTA's imaging plane. The USTA's output is directly connected to a 512‐channel 26‐dB preamplifier to reduce the cable length and electrical noise. The amplified acoustic signal is digitized by four 128‐channel data acquisition systems (DAQs) (SonixDAQs, Ultrasonix Medical, Corp), stored in the onboard flash memory, and then transferred to a personal computer via the universal serial bus. The DAQs are configured to sample at 40 MHz, with a 20‐MHz analog low‐pass filter, 12‐bit dynamic range, and programmable amplification up to 51 dB. The USTA and SFUST are enclosed in a negatively pressurized deionized water chamber sealed by an optically and acoustically transparent plastic membrane. By reducing the chamber pressure, we deform the membrane into a concave shape to accommodate the head geometry, minimizing the water downforce on the head. Colorless ultrasound gel (Aquasonic Clear, Parker Laboratories, Inc.) is applied between the head and the membrane to enhance acoustic coupling. PAT uses two laser wavelengths to quantify the oxygen saturation (sO_2_) and hemoglobin concentration changes: 1) a 532‐nm Nd:YAG laser connected to a second harmonic module; ≈5‐nm pulse width, Brilliant B, Quantel, Inc., and 2) a 594‐nm optical parametric oscillator laser; ≈5–8‐nm pulse width, SpitLight EVO III, Innolas, GmbH. A beam sampler (BSF20‐A, Thorlabs, Inc.) partially reflects the laser beam to a photodiode (DET36A, Thorlabs, Inc.) to correct for the pulse‐to‐pulse energy fluctuation. The laser beams are spatially aligned before entering the engineered optical diffuser (EDC‐20, RPC, Inc.).

Figure 1b is a cutaway view of the CRUST‐PAT. The overlaid laser and ultrasound excitation fields form an imaging FOV ≈1 cm in diameter and meet the spatial Nyquist sampling criteria.^[^ ^14^ ^]^ The SFUST can be simplified to a virtual point source (VPS) firing spherical ultrasonic waves. In addition to the crossed transmission and detection fields that offer angle‐independent flow measurements, the panoramic detection aperture gives these measurements omnidirectional sensitivity, distinguishing CRUST from conventional Doppler ultrasound imaging. For example, in Figure 1c, although the flow velocity in the *xz* plane has zero projections on the *y*‐axis, its projections on the excitation and other detection rays are nonzero. For flow velocity with equal projection coefficients along the excitation and detection rays, its projections on other detection rays are nonzero, meaning that the Doppler frequency shift can consistently be detected regardless of the flow direction. In contrast, ultrafast ultrasound and conventional Doppler ultrasound imaging detect partial flow velocity components and therefore partial Doppler frequency shift, resulting in reduced sensitivity for transverse flow due to the small or zero velocity projections on the acoustic axes, as demonstrated in Figure 1d. The difference between CRUST and conventional Doppler ultrasound imaging is illustrated in Video S1, Supporting Information.

To facilitate simultaneous CRUST and PAT acquisition, the triggers of the SFUST and the laser system are interleaved, as shown in Figure 1e. The PRF of CRUST is tunable up to 20 kHz. To avoid acoustic cross talk from sound reverberation in the chamber, we used a 4‐kHz PRF, corresponding to 392 pulses in a pulse train. The interval between the laser pulses and CRUST pulse trains is 0.5 ms.

### 2.2 CRUST Image Reconstruction

**Figure 2:**
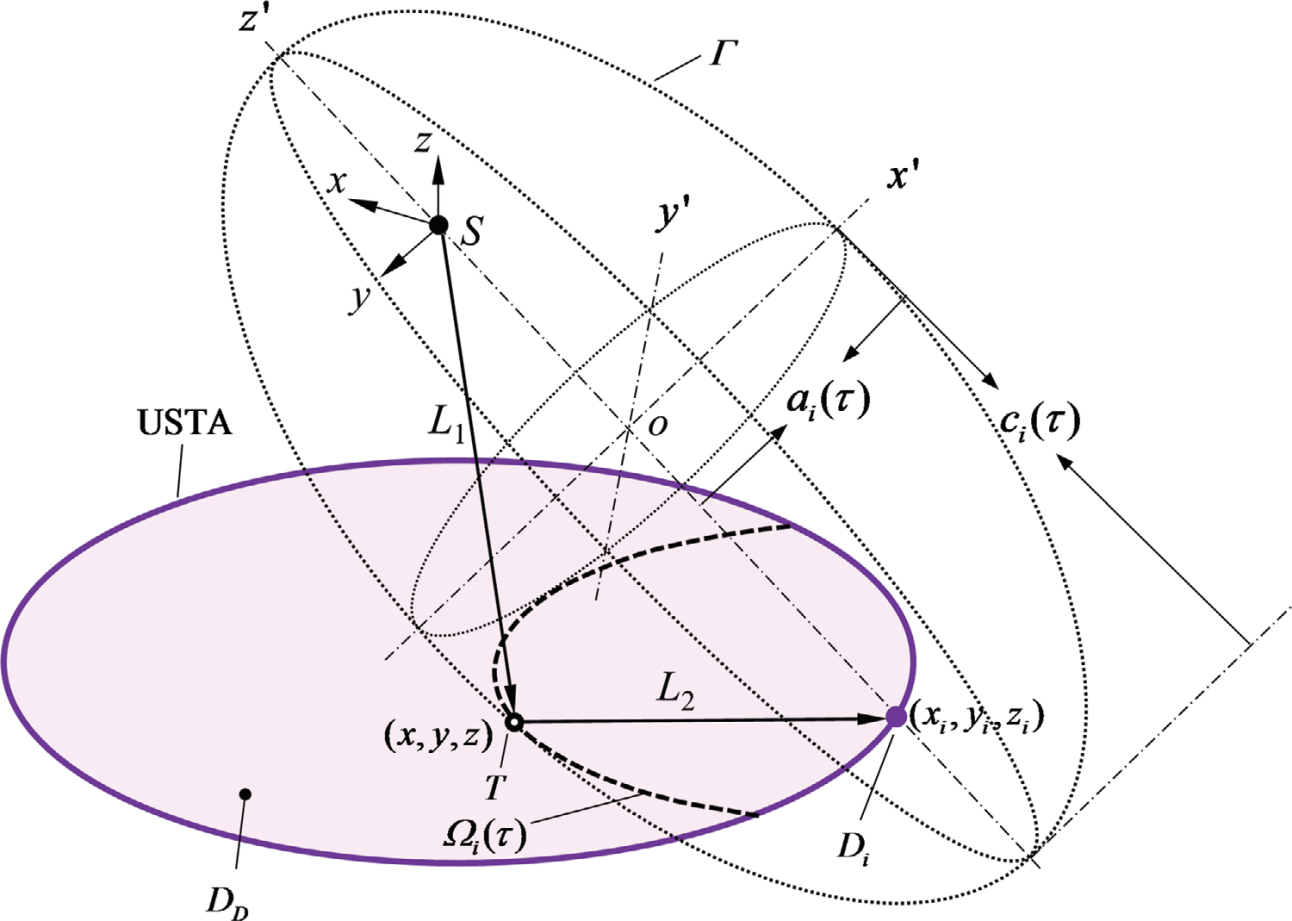
Schematic of CRUST image reconstruction. *S*, the VPS position and the origin of the global coordinate system *S*‐*xyz*; *D_D_*, the imaging plane defined by the USTA; *D_i_*, the *i‐*th element position of the USTA at (*x_i_*, *y_i_*, *z_i_*); *T*, a point scatterer position in *D_D_* at (*x*, *y*, *z*); *L*_1_, the distance between *S* and *T*; *L*_2_, the distance between *T* and *D_i_*; *τ*, the time of flight along *L*_1_ and *L*_2_; *c*, the speed of sound; Γ, a spheroid originating at *o* with its symmetric axis along the *y*′‐axis of the coordinate system *o*‐*x*′*y*′*z*′; *a_i_* (*τ*), the equatorial radius of Γ; *c_i_* (*τ*), the distance between the center of Γ and the pole on the symmetric axis. Ω_*i*_(*τ*), a partial ellipse formed by the intersection of *D_D_* and Γ.

CRUST solves an inverse problem to form widefield images. In **Figure** **2**, we define a global coordinate system *S*‐*xyz* with the VPS position as the origin, the imaging plane as *D_D_*, the *i‐*th element position of the USTA as *D_i_* at (*x_i_*, *y_i_*, *z_i_*), and a point scatterer position in *D_D_* as *T* at (*x*, *y*, *z*). We denote the distance between *S* and *T* as *L*_1_, the distance between *T* and *D_i_* as *L*_2_, the acoustic time of flight along *L*_1_ and *L*_2_ as *τ*, and the speed of sound as *c*.

From the viewpoint of *D_i_*, the distribution of *T* yields a partial ellipse Ω_*i*_(*τ*) for each value of *τ*, which is represented by the intersecting line of *D_D_* and a spheroid Γ in the form of

(1) $$\begin{array}{ccc} & & \frac{1}{a_{i}{\left(\tau \right)}^{2}}\left(x^{2}+y^{2}+z^{2}-\frac{{\left(xx_{i}+yy_{i}+zz_{i}\right)}^{2}}{SD_{i}^{2}}\right)\\ & & \;+\frac{1}{c_{i}{\left(\tau \right)}^{2}}\;{\left(\frac{xx_{i}+yy_{i}+zz_{i}}{SD_{i}}-\frac{SD_{i}}{2}\right)}^{2}=\;1 \end{array}$$

Here $a_{i}\left(\tau \right)=1/4{\left({\left(c\tau \right)}^{2}-SD_{i}^{2}\right)}^{1/2}$ refers to the equatorial radius, and *c_i_* (*τ*) = *cτ*/2 is half the distance between the poles on the symmetric axis. Assuming an infinite‐bandwidth electrical impulse response, electrical input in the form of a *δ* function, and Rayleigh scattering, we express the acoustic pressure observed by *D_i_* at *t* = *τ* as the ellipsoidal Radon transform:

(2) $$p_{i}\;\left(t\right)=\frac{1}{4\pi c^{2}}\;\frac{\partial }{\partial t}\left(\frac{1}{ct-L_{1}}\int_{\Omega_{i}\left(\tau \right)}P_{0}\left(x,y,z\right)dxdy\right)$$

Here, *P*_0_ denotes the amplitude of the scattered pressure. Because *T* is defined in *D_D_*, *z* is constant. To retrieve *P*_0_, we can solve the inverse ellipsoidal Radon transform, whose solution is exact when *p* is measurable on a spherical surface enclosing the scatterers.^[^ ^23^ ^]^ For CRUST, *P*_0_ can be estimated using the inverse ellipsoidal Radon transform with detectors in the imaging plane:

(3) $$\begin{array}{ccc} & & P_{0}\left(x,y,z\right)\approx \frac{1}{\sum_{i\;=\;1}^{N}\psi_{i}\left(x,y,z\right)}\\ & & \;\sum_{i\;=\;1}^{N}\left[\left(2p_{i}\left(\tau \right)-2\left(t-\frac{L_{1}}{c}\right)\frac{\partial p_{i}\left(t\right)}{\partial t}{|}_{t\;=\;\tau }\right)\psi_{i}\left(x,y,z\right)\right] \end{array}$$

where *N* stands for the USTA's total element count, and *ψ*_*i*_(*x*,*y*, *z*) represents the planar angle for *D_i_* with respect to *T*. Since (*t* − *L*_1_/*c*)∂*p_i_* (*t*)/∂*t* ≫ *p_i_* (*t*) holds valid for typical medical ultrasound frequencies, *p_i_* (*τ*) in Equation (3) can be omitted, simplifying Equation (3) to the form of filtered back projection.^[^ ^23^ ^]^ Due to the limited bandwidth, a realistic ultrasonic detector usually behaves similarly to a ramp filter in the frequency domain. As a result, ∂*p_i_* (*t*)/∂*t* can be approximated as the measured pressure, further simplifying Equation (3) to

(4) $$P_{0}\left(x,y,z\right)\approx \frac{\sum_{i\;=\;1}^{N}\left[2p_{i}\left(\tau \right)\left(\tau -\frac{L_{1}}{c}\right)\psi_{i}\left(x,y,z\right)\right]}{\sum_{i\;=\;1}^{N}\psi_{i}\left(x,y,z\right)}$$

In the following, we denote the pixel value of a CRUST image as *A*. It is related to *P*_0_ by the system's proportionality coefficient, that is, a constant ratio between the recorded digital signal and the detected acoustic pressure.

**Figure 3:**
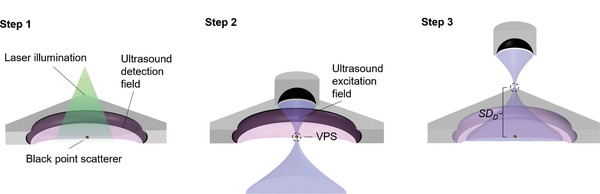
CRUST calibration.

To implement the reconstruction algorithm, we first calibrated the system using the method illustrated in **Figure** **3**. A point scatterer made of stainless steel, coated with black dye, and embedded in agar, was used as the calibration sample. In step 1, the sample was positioned approximately at the array's center and moved along the elevational direction while being imaged by PAT to identify the USTA's focal plane. The point scatterer's in‐plane coordinates were estimated based on the PAT images. In step 2, the SFUST was operated in pulse‐echo mode and scanned horizontally until the VPS coincided with the point scatterer. In Step 3, the SFUST was displaced by *SD_D_* in the elevational direction to yield a desired FOV in the imaging plane.

**Figure 4:**
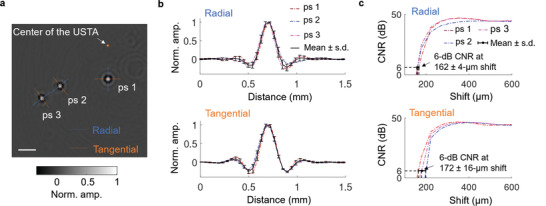
Characterization of CRUST. a) Reconstructed acoustic point scatterers located at different distances from the USTA's center. The image is normalized to the maximum pixel value and presented in real values. b) Amplitude distributions along the radial and tangential line profiles in (a). The curves are normalized to the maximum values to facilitate comparison. c) CNR versus the shift in the sum of each original line profile in (b) and the shifted one. The contrast is defined as the average amplitude difference between the crest and the trough of the summed profile over 28 repeated measurements. The single‐measurement noise is defined as the root‐mean‐square of the amplitude standard deviations at the crest and trough across 28 repeated measurements. Scale bar = 1 mm; Norm. = normalized; Amp. = amplitude.

After system calibration, we imaged three point scatterers embedded in agar and randomly distributed in the FOV. **Figure** **4**a displays the reconstructed image. The amplitude distributions along the radial and tangential line profiles, which go through the highest‐value pixels, are plotted in Figure 4b. The contrast‐to‐noise ratio (CNR) versus the shift in the sum of the original profile and the shifted one is displayed in Figure 4c. The radial and tangential resolutions, defined by the shifts corresponding to a 6‐dB CNR, were quantified to be 162 ± 4 and 172 ± 16 µm, respectively.

### 2.3 Power Doppler Imaging

Defining *A*(*j*) as the pixel value of the *j*‐th image, we first applied clutter filtration based on singular value decompensation to remove the tissue background.^[^ ^25^, ^26^ ^]^ The pixel value of the *j*‐th filtered image is represented by *A*′(*j*). We generated the power Doppler images (PDIs) by taking the average intensity of each pixel over *m* filtered images:

(5) $$I_{\mathrm{PDI}}=\frac{1}{m}\;\sum_{j\;=\;1}^{m}A^{\prime }{\left(j\right)}^{2}$$

**Figure 5:**
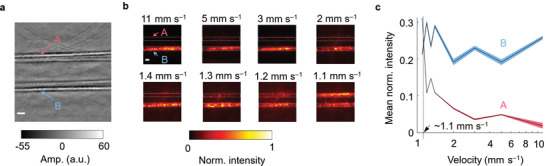
PDIs of CRUST. a) Structural image of two tubes filled with static (A) and flowing (B) BMF. b) PDIs of the BMF at various flow velocities. The images are normalized to the maximum pixel intensities. c) Mean PDI intensity calculated in the flow regions versus the flow velocity. The shadow areas represent 95% confidence intervals (assuming a normal distribution), corresponding to ±1.96 times the standard errors. Scale bars = 1 mm; Norm. = normalized; Amp. = amplitude.

We evaluated CRUST by imaging BMF (CAE, Inc.) at various flow velocities in a 0.6‐mm‐inner‐diameter silicone tube. Another identical tube filled with static BMF was used as the negative control. Structural images of the two tubes are shown in **Figure** **5**a. Figure 5b displays the PDIs produced from 98 images acquired at 1 kHz in 100 ms. For the phantom studies, we removed the first ten eigenvectors. Figure 5c shows the averaged pixel intensities in the flow regions at different flow velocities. The crossover indicates a minimum detectable flow velocity of ≈1.1 mm s^−1^. Since the imaging sensitivity is proportional to the square root of the total number of images, a lower flow velocity is expected to be measured with a longer acquisition time.^[^ ^9^ ^]^

### 2.4 Vector Flow Estimation

**Figure 6:**
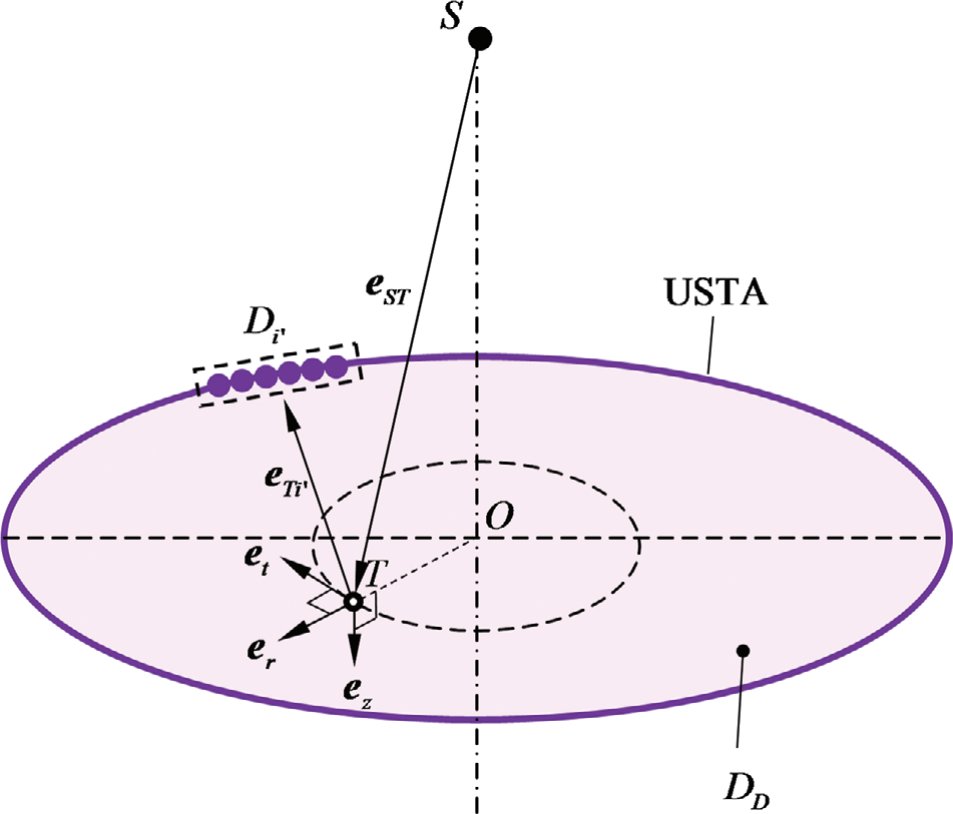
Schematic of vector flow estimation using CRUST.

In **Figure** **6**, we define *O* as the projection of *S* on *D_D_*, *e_r_* as the radial unit vector pointing from *O* to *T*, *e_t_* as the tangential unit vector originating at *T* and perpendicular to *e_r_*, and *e_z_* as the vertical unit vector originating at *T* and perpendicular to *D_D_*. *e_r_*, *e_t_*, and *e_z_* are along the first, second, and third axes of a right‐handed coordinate system, respectively. The velocity vector *v* at *T* can be expressed as *v* = *v_r_e_r_* + *v_t_e_t_* + *v_z_e_z_*, where *v_r_*, *v_t_*, and *v_z_* are the projection coefficients.

We define *D*_*i*′_ as the *i*′‐th subgroup of the USTA elements and *A*_*i*′_(*j*) as the pixel value of the *j*‐th frame reconstructed with *D*_*i*′_. We assign 128 elements to *D*_*i*′_ to improve its reconstruction quality and used a 64‐element sliding window to define *D*_*i*′ + 1_, resulting in a total *I*′ = 8 subgroups of the USTA elements. Denoting the unit vector from *S* to *T* as *e_ST_*, the unit vector from *T* to the center of *D*_*i*′_ as *e*_*Ti*′_, and the carrier frequency of the transmission ultrasound as *f*_0_, the Doppler frequency shift at *T* viewed by *D*_*i*′_ can be expressed as

(6) $$\begin{array}{ccc} f_{Ti^{\prime }} & = & \frac{f_{0}}{c}\;\left(-v\cdot e_{ST}+v\cdot e_{Ti^{\prime }}\right)\\ & = & \frac{f_{0}}{c}\;\left[v_{r}e_{r}\cdot \left(-e_{ST}+e_{Ti^{\prime }}\right)+v_{t}e_{t}\cdot e_{Ti^{\prime }}-v_{z}e_{z}\cdot e_{ST}\right] \end{array}$$

Equation (6) holds valid for |*v*| ≪ *c* and can be rewritten in a matrix notation as

(7) $$\left[\begin{array}{ccc} e_{r}\cdot \left(-e_{ST}+e_{T1}\right) & e_{t}\cdot e_{T1} & -e_{z}\cdot e_{ST}\\ \vdots & \vdots & \vdots \\ e_{a}\cdot \left(-e_{ST}+e_{Ti^{\prime }}\right) & e_{t}\cdot e_{Ti^{\prime }} & -e_{z}\cdot e_{ST}\\ \vdots & \vdots & \vdots \\ e_{a}\cdot \left(-e_{ST}+e_{TI^{\prime }}\right) & e_{t}\cdot e_{TI^{\prime }} & -e_{z}\cdot e_{ST} \end{array}\right]\;\left[\begin{array}{c} v_{r}\\ v_{t}\\ v_{z} \end{array}\right]=\left[\begin{array}{c} u_{1}\\ \vdots \\ u_{i^{\prime }}\\ \vdots \\ u_{I^{\prime }} \end{array}\right]$$

where

(8) $$u_{i^{\prime }}=\frac{cf_{Ti^{\prime }}}{f_{0}}$$

Equation (7) describes an overdetermined problem with *f*_*Ti*′_ as the input and *v_r_*, *v_t_*, and *v_z_* as the three unknowns. For each pixel, clutter filtering was first applied to the image ensemble to remove the tissue background, and the pixel value of the *j*‐th filtered image is denoted by *A*′_*i*′_(*j*). We retrieved a pixel's amplitude and temporal frequency from its analytic signal $\bar{A{{}^{\prime }}_{i^{\prime }}}\left(j\right)$. *f*_*Ti*′_ was computed through lag‐one autocorrelation, using the analytic signal in *m* successive frames.^[^ ^27^ ^]^ The lag‐one autocorrelation is defined as

(9) $$\Upsilon_{i^{\prime }}\;\left(1\right)=\sum_{j=1}^{m}\bar{{A^{\prime }}_{i^{\prime }}}\left(j\right)\mathrm{conj}\left(\bar{{A^{\prime }}_{i^{\prime }}}\left(j-1\right)\right)$$

where conj() represents the complex conjugate. *f*_*Ti*′_ was found using *f*_*Ti*′_ = *f_s_* tan^‐1^(Im(ϒ_*i*′_(1))/Re(ϒ_*i*′_(1)))/(2*π*), where *f_s_* is the FR. Denoting the coefficient matrix (first term) of Equation (7) as *H* and the last term as *u*, we solved the coefficient vector using least‐squares fitting:

(10) $$\left[\begin{array}{c} v_{r}\\ v_{t}\\ v_{z} \end{array}\right]=\;{\left(H^{T}H\right)}^{-1}H^{T}u$$

**Figure 7:**
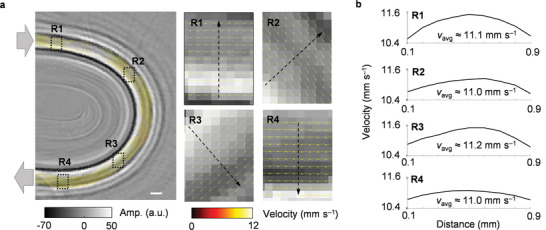
Ex vivo vector flow estimation by CRUST. a) Velocity map overlaid on the structural image of a U‐shaped tube. R1 and R4 refer to the straight‐tube regions near the entrance and exit. R2 and R3 represent the bent‐tube regions. Magnified flow velocity vectors in R1–R4 are shown on the right. b) Velocity amplitude distributions along the line profiles in R1–R4. The computed average velocities are denoted by *v*_avg_. Scale bars = 1 mm; Amp. = amplitude.

We evaluated CRUST for vector flow estimation by imaging BMF at a controlled flow velocity of 11 mm s^−1^ in a U‐shaped tube with a 1‐mm inner diameter. Vector flow was estimated based on lag‐one autocorrelation and least‐squares fitting, using 98 images acquired in 100 ms.^[^ ^27^ ^]^ **Figure** **7**a shows the estimated velocity map and magnified velocity vectors. Figure 7b displays the velocity distributions along the line profiles in the straight‐tube regions (R1 and R4) and the bent‐tube regions (R2 and R3). Laminar flow with a symmetric velocity distribution along the central axis is observed in R1 and R4. In R2 and R3, the faster‐moving BMF tended to deviate from the tube's center toward the outer region of the bend. Given the approximately uniform sideways pressure gradient, which resulted in uniform sideways acceleration on the unit mass across the tube's cross section, the faster‐moving BMF changed its direction less rapidly than the slower‐moving BMF due to the larger momentum.^[^ ^28^ ^]^

## 3 Results

### 3.1 Functional Study Protocol

**Figure 8:**
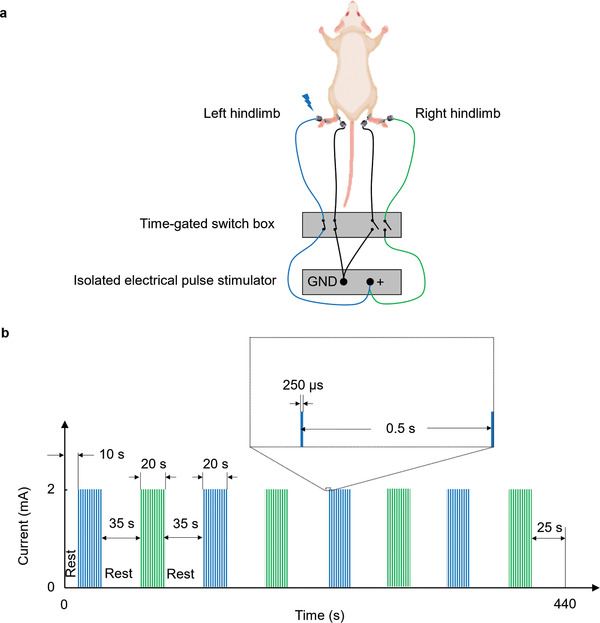
Bilateral hindlimb electrical stimulation. a) Schematic of the electrical simulation setup. Left and right hindlimb stimulation was alternately applied using the time‐gated switch box. Shown is stimulation to the left hindlimb. b) Electrical stimulation paradigm.

In the functional study, hindlimb electrical stimulation was implemented using two pairs of micro clip electrodes (Model RS‐5420, Roboz Surgical Instrument, Co.) extended from an isolated electrical pulse stimulator (Model 2100, A‐M Systems, Inc.). The two electrode pairs were isolated by an electrical switch box synchronized with the imaging system, as illustrated in **Figure** **8**a. The periodic stimulation consisted of eight stimulation cycles; each included a 20‐s stimulation period followed by a 35‐s resting period (a 10‐s resting period in the beginning and a 25‐s resting period following the last stimulation period). Each stimulation period consists of 40 unipolar pulses with a 2‐Hz PRF, 2‐mA current amplitude, and 250‐µs pulse width (Figure 8b). The stimulation period and intensity were carefully controlled without inducing noticeable motion.

### 3.2 CRUST‐PAT of the Mouse Brain with the Intact Scalp and Skull

**Figure 9:**
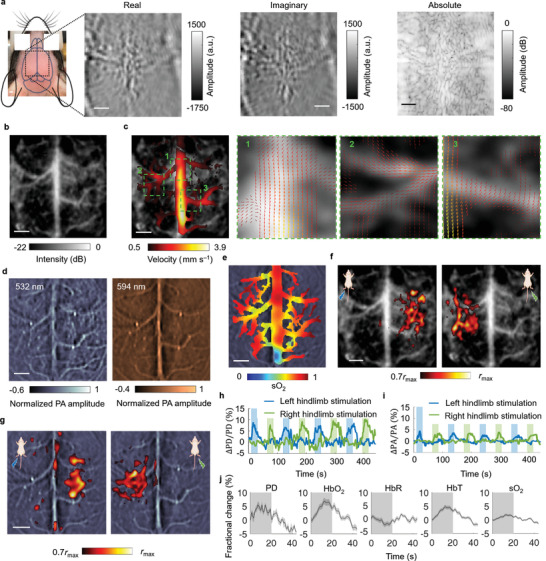
CRUST‐PAT of the mouse brain with the intact scalp and skull. a) Widefield CRUST images displayed with real, imaginary, and absolute pixel values. b) PDI of the mouse brain. c) Velocity amplitude map of the CBF in the major cortical vessels (left). Flow vectors in regions 1–3 are magnified and displayed on the right. d) Widefield PAT images acquired at 532‐ and 594‐nm optical wavelengths. The images are normalized to the maximum pixel values. e) sO_2_ map estimated using the widefield PAT images acquired at the two wavelengths. f) CRUST‐measured functional responses presented using the Pearson correlation coefficients thresholded at 70% of the maximum (*r*_max_), showing contralateral functional responses to the hindlimb electrical stimulation. g) PAT‐measured functional maps presented in the same way as (f). h) Functional signal of CRUST. The signal represents the moving average (temporal window size of 4 s) of the mean values of the activated pixels in (f). i) Functional signal of PAT presented in the same way as (h). j) Computed fractional changes of PD, hemoglobin concentrations, and sO_2_ signal in response to stimulation. Data are mean ± s.e.m., *n* = 8 stimulation cycles, technical replicates. For scale bars = 1 mm.

**Figure 10:**
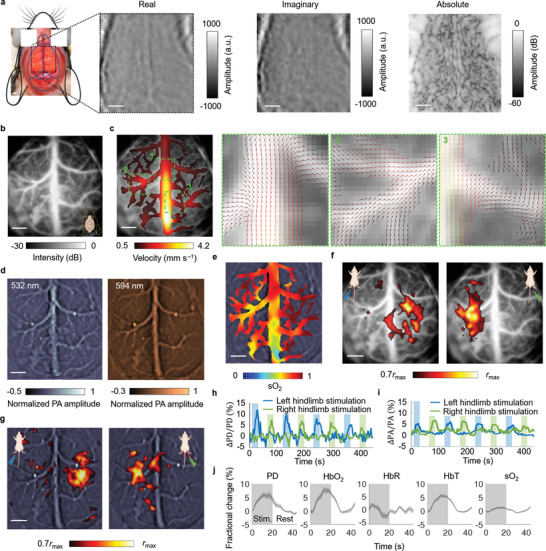
CRUST‐PAT of the mouse brain with the scalp and skull removed. a) Widefield CRUST images displayed with real, imaginary, and absolute pixel values. b) PDI of the mouse brain. c) Velocity amplitude map of the CBF in the cortical vessels. Regions 1–3 are magnified, showing detailed flow vectors at the blood vessel bifurcations. d) PAT images of the mouse brain acquired at 532‐ and 594‐nm optical wavelengths. The images are normalized to the maximum pixel values. e) sO_2_ map estimated using the PAT images acquired at the two wavelengths. f) CRUST‐measured functional responses presented using the Pearson correlation coefficients thresholded at 70% of the maximum, showing contralateral functional responses to the hindlimb electrical stimulation. g) PAT‐measured functional maps presented in the same way as (f). h) Temporal functional signal of CRUST, showing contralateral functional responses. The signal represents the moving average (4‐s temporal window size) of the mean values of the activated pixels in (f). i) PAT's temporal functional signal presented in the same way as (h), showing contralateral responses to the hindlimb electrical stimulation. j) Computed fractional changes of PD, hemoglobin concentrations, and sO_2_ signal in response to stimulation. Data are mean ± s.e.m., *n* = 8 stimulation cycles, technical replicates. For scale bars = 1 mm.

The CRUST images were acquired at a 4‐kHz PRF, resulting in an FR of 200 Hz after 20 times averaging. The 200‐HZ FR allows a maximum flow velocity of ≈2.8 cm s^−1^ to be measured (based on Equation (8), |*u*_*i*′max_| = *cf*_*Ti*′max_/*f*_0_, where *c* ≈ 1500 m s^−1^, *f*_*Ti*′max_ = FR/2 = 100 Hz, and *f*_0_ = 5.4 MHz). **Figure** **9**a displays the CRUST images, and Figure 9b shows the PDI acquired through the intact scalp and skull in 1 s. In this study, the first 18–30 vectors were removed, depending on the degree of tissue motion. Indeed, compared to the post‐craniectomy PDI (to be shown in **Figure** **10**b), the image quality of Figure 9b is considerably degraded because of the skull‐induced two‐way acoustic aberration, including attenuation and distortion.^[^ ^29^ ^]^ Nevertheless, major cortical vessels are resolved, owing to the relatively thin skull thickness (≈280 ± 40 µm, averaged over four locations) and the moderate ultrasound frequency used (corresponding to a ≈280‐µm wavelength at the central frequency). Figure 9c displays the computed velocity amplitudes in the cortical vessels overlaid on the PDI. Magnified CBF vectors at different locations, where blood vessel bifurcations are present, are displayed on the right. The flow patterns agree well with the known cerebral circulation, in which the blood flow in the venules merges in the veins and then drains into the superior sagittal sinus.^[^ ^30^ ^]^ PAT images were acquired simultaneously, using the interlaced excitation sequences described in Figure 1e. Figure 9d shows the PAT images acquired at 532‐ and 594‐nm optical wavelengths and averaged over ten frames obtained in 1 s.

We used CRUST‐PAT to image task‐evoked brain activities in response to the bilateral hindlimb electrical stimulation. CRUST and PAT determined regions of brain activities based on their increased blood volume and hemoglobin concentrations due to neurovascular coupling. The reconstructed PDI and PAT image sequences were first spatially smoothed with a Gaussian kernel (0.3‐mm full width at half maximum) to enhance the signal‐to‐noise ratio (SNR) and then passed through a high‐pass filter (0.01 Hz, fourth‐order Butterworth) to remove the long‐term drift. The resultant pixel values were added to the average pixel values of the original images to maintain their magnitudes. Figure 9e shows the baseline oxygen saturation (sO_2_) estimated based on linear spectral fitting in the blood vessel regions.^[^ ^31^, ^32^ ^]^ Defining *C* as the molar concentration, *ε* as the molar extinction coefficient,^[^ ^32^ ^]^ *P* ^PA^ as the reconstructed PA amplitude, *K* as the system's proportionality coefficient relating *P* ^PA^ to the absorption coefficient, and *φ* as the local optical fluence, we describe the linear spectral fitting as^[^ ^31^ ^]^

(11) $$\begin{array}{ccc} \left[\begin{array}{c} C_{{\mathrm{HbO}}_{2}}\\ C_{\mathrm{HbR}} \end{array}\right] & = & {\left(\varepsilon^{T}\varepsilon \right)}^{-1}\;\varepsilon^{T}P^{\mathrm{PA}}K,\;P^{\mathrm{PA}}=\left[\begin{array}{c} \frac{P_{594}^{\mathrm{PA}}}{\phi_{594}}\\ \frac{P_{532}^{\mathrm{PA}}}{\phi_{532}} \end{array}\right],\\ \varepsilon & = & \left[\begin{array}{cc} \varepsilon_{{\mathrm{HbO}}_{2}\_594} & \varepsilon_{\mathrm{HbR}\_594}\\ \varepsilon_{{\mathrm{HbO}}_{2}\_532} & \varepsilon_{\mathrm{HbR}\_532} \end{array}\right] \end{array}$$

sO_2_ is defined as ${\mathrm{sO}}_{2}=C_{{\mathrm{HbO}}_{2}}/\left(C_{{\mathrm{HbO}}_{2}}+C_{\mathrm{HbR}}\right)$. Since *K* is unknown, only relative concentrations are computed.

Figure 9f presents the CRUST‐measured activation map using the Pearson correlation coefficients between the power Doppler (PD) signal and the temporal stimulation patterns. The molar extinction coefficient of deoxyhemoglobin (HbR) diverges more from that of oxyhemoglobin (HbO_2_) at 594 nm than at 532 nm ($\varepsilon_{\mathrm{HbR}\_594}\approx 3\varepsilon_{{\mathrm{HbO}}_{2}\_594}$; $\varepsilon_{\mathrm{HbR}\_532}\approx \varepsilon_{{\mathrm{HbO}}_{2}\_532}$; *ε*: molar extinction coefficient), and we used the PA signal acquired at 594 nm to derive the activation map for PAT in Figure 9g. The achieved functional distributions of CRUST and PAT demonstrate that the hindlimb stimulation caused signal changes in the contralateral primary somatosensory barrel cortex (S1). We further spatially averaged the temporal signal across the activated pixels for the stimulation on each side. Figure 9h displays the temporal signal of CRUST, showing a maximum PD signal increase of ≈11%. The changes in the CBF velocities in response to the electrical stimulation are displayed in Video S2, Supporting Information. In comparison, the temporal signal of PAT in Figure 9i indicates a maximum PA signal increase of ≈5%. For both CRUST and PAT, the silence of the signal in the homolateral brain hemisphere during hindlimb stimulation demonstrates the spatial specificity of CRUST‐PAT. Figure 9j shows the fractional changes of the PD, hemoglobin concentration, and sO_2_ signal averaged over eight stimulation cycles. Defining the onset time as the time to 50% peak (>2 s.e.m. for the baseline), we found CRUST to exhibit a faster response (≈4.1 s) than PAT (≈6.2 s for HbT and ≈7.1 s for sO_2_). This observation highlights the benefit of merging the two modalities, which complement each other in both contrasts and detection speed.

Overall, CRUST‐PAT has demonstrated its capability of acquiring vector flow and sO_2_ information through the intact scalp and skull with a large FOV and considerable vasculature detail at some expense of image quality. On the other hand, imaging through the scalp and skull is generally challenging for most label‐free optical flow imaging methods, including Doppler‐based laser flowmetry, RBC‐tracking‐based laser scanning microscopy, and speckle‐based imaging.

### 3.3 CRUST‐PAT of the Mouse Brain with the Scalp and Skull Removed

We repeated the measurements on the same mouse after removing the scalp and skull. Figure 10a displays the CRUST images, and Figure 10b shows the PDI acquired using the identical sequences as Figure 9b. As expected, the image quality of Figure 10b is improved due to the absence of the skull‐induced acoustic aberration.

Figure 10c shows the flow velocity map and magnified flow vectors in the major cortical vessels. Acceptable agreement between the velocity distributions in Figures 9c and 10c can be found, but the distorted or undetected flow vectors at some locations in Figure 9c are better revealed in Figure 10c. Figure 10d shows PAT images acquired at 532‐ and 594‐nm optical wavelengths and averaged across ten frames. Compared to CRUST, the PAT images in Figure 9d retain more details in Figure 10d, owing to PAT's one‐way acoustic aberration and the limited light attenuation induced by the scalp and skull. Figure 10e shows the sO_2_ distribution computed in the same cortical vessel regions as Figure 9e. Figures 10f and 10g display the CRUST and PAT functional maps, respectively. Contralateral responses to the hindlimb stimulation are revealed in the S1 cortex. The spatially averaged temporal signal shows a maximum PD signal increase of ≈12% (Figure 10h) and a maximum PA signal increase of ≈7% (Figure 10i), agreeing well with the results in Figure 9. Video S3, Supporting Information, displays the changes in the CBF velocities in response to the electrical stimulation for the trepanned mouse. Figure 10j shows the functional changes of hemoglobin concentrations and sO_2_ averaged across eight stimulation cycles. The onset time of CRUST (≈3.9 s) also indicates a faster response than PAT (≈5.8 s for HbT and ≈7.3 s for sO_2_), agreeing with Figure 9.

## 4 Discussion

The principle of CRUST can be extended to a more general ultrasound‐imaging concept: using spherical acoustic waves for widefield excitation and cross‐axis detectors for detection.^[^ ^24^ ^]^ Figure S1, Supporting Information, illustrates possible transmission and detection configurations. For transmission, either a real or a VPS can be employed to send the spherical excitation waves. A real point source can be a PA‐based passive absorber or a tiny single‐element transducer with a diameter smaller than half the acoustic wavelength. For detection, a 1D linear array can be used for 2D imaging. A 2D linear array, cylindrical array, or spherical array can be employed for 3D imaging.

We have presented CRUST‐PAT—a hybrid imaging method offering both blood flow information enabled by CRUST and molecular specificity provided by PAT. Without hardware modification, CRUST‐PAT was realized by adding an SFUST to an established full‐ring‐shaped PAT system. Unlike conventional ultrasonic transducer probes, the full‐ring‐shaped transducer array equips CRUST with omnidirectional sensitivity to the in‐plane CBF and nearly isotropic resolution in the FOV. For brain imaging, the time of hemodynamic response to single‐impulse stimulation has been reported to be as short as 130 ms in a single capillary.^[^ ^33^ ^]^ Nevertheless, the response time on the coarser scale of capillary beds or cortical veins and cerebral venous sinuses has been measured to be on the order of several seconds.^[^ ^9^, ^12^, ^15^, ^34^, ^35^ ^]^ Therefore, we used an acquisition time of 1 s to produce the PDI and CBF vectors with CRUST. Currently, the CRUST‐PAT system does not support real‐time image reconstruction due to the DAQ architecture, that is, the combination of flash memory and offline data transfer. Nevertheless, the architecture makes data acquisition at an ultrahigh PRF possible, benefitting the maximum detectable velocity and averaging times for SNR enhancement. Compared with conventional pulse‐echo ultrasound imaging that uses a focused transducer or digital beamforming, the unfocused wave transmission of ultrafast ultrasound and CRUST does not provide in‐plane transmission focusing. While ultrafast ultrasound provides elevational resolution in transmission, CRUST does so through the detection aperture. Due to the relatively flat contour of the mouse's cerebral cortex, out‐of‐focus problems were mitigated by using PAT to assist the alignment. Since an RBC acts as a Rayleigh scatterer at ultrasound frequencies below ≈18 MHz, finer spatial resolution can be obtained using a higher ultrasound frequency.^[^ ^36^ ^]^ However, a higher frequency may reduce the FOV, increase acoustic attenuation in water and soft tissues, and increase acoustic aberration induced by the skull.^[^ ^37^ ^]^ Alternatively, contrast agents, such as microbubbles, can be employed to improve the resolution and sensitivity.^[^ ^20^, ^38^ ^]^

The ability to image CBF and sO_2_ at high spatiotemporal resolution using CRUST‐PAT could be of great interest for applications where conventional optical imaging methods reach their limits, for example, imaging epileptic‐induced changes of CBF and sO_2_ simultaneously.^[^ ^39^ ^]^ CRUST‐PAT could potentially be implemented on a miniaturized PAT device to facilitate comprehensive characterization of the cerebral hemodynamics in the brains of behaving animals.^[^ ^40^ ^]^ Although imaging the human brain transcranially using the current CRUST‐PAT configuration is challenging due to the skull‐induced acoustic aberration, recent advances in microbubble‐aided localization microscopy and human brain PAT have demonstrated potential, given that USTAs of a low central frequency (≈1 MHz) and more powerful de‐aberration algorithms are available.^[^ ^11^, ^12^, ^41^ ^]^

## 5 Experimental Section

### Animal Preparation

Adult 7‐week‐old mice (C57, 25–30‐g body weight, male, Charles River Laboratories, Inc.) were imaged using CRUST‐PAT. Maintained under anesthesia with 2% vaporized isoflurane, the mice were placed on a lab‐made holder adjustable in six degrees of freedom. The body temperature was maintained by a feedback‐controlled heating pad on the holder. The head was stabilized using a nose cone, a tooth bar, and two ear bars. Before imaging, the hair on the head and hindlimbs was removed using clippers and depilatory cream. After the mouse was transferred to the imaging system, the isoflurane level was decreased to 0.6% to awaken the brain. Functional imaging was first conducted with the intact scalp and skull. Then the mouse was transferred to a surgery table to remove the scalp and skull, after which the mouse was moved back to the imaging system to repeat the experiments. All animal imaging experiments were performed following relevant guidelines and regulations and approved by the Institutional Animal Care and Use Committee of the California Institute of Technology.

### Statistical Analysis

Raw channel data downloaded from the DAQs were corrected for the jitter using a reference impulse interference signal. The corrected data were then used to reconstruct the PDI and PAT image sequences, which were spatially smoothed to enhance the SNR and passed through a high‐pass filter to remove the long‐term drift before being used for the in vivo functional studies. The experimental data in Figures 9j and 10j were presented as mean ± s.e.m., and the sample size *n* = 8 represented technical replicates. Image reconstruction and data analysis were carried out using MATLAB.

## Conflict of Interest

L.V.W. has a financial interest in Microphotoacoustics, Inc., CalPACT, LLC, and Union Photoacoustic Technologies, Ltd., which, however, did not support this work.

## Authors Contribution

S.N. and Y.Z. contributed equally to this work. L.V.W. and S.N. developed the CRUST method. S.N. developed the system and reconstruction software. S.N. and Y.Z. performed the experiments. L.V.W. proposed the original CRUST concept and supervised the study. S.N. wrote the manuscript with inputs from all authors.

## Supporting information

Supporting Information

Supplemental Video 1

Supplemental Video 2

Supplemental Video 3

## Data Availability

The data that support the findings of this study are provided within the paper and its Supporting Information.
