# Supplementary material for: Cross‐Ray Ultrasound Tomography and Photoacoustic Tomography of Cerebral Hemodynamics in Rodents
Source: Adv Sci (Weinh). 2022 Jul 11;9(25):2201104. doi: 10.1002/advs.202201104 (PMC9443457; doi:10.1002/advs.202201104)
Supplement: Supplementary file 1 — Supporting Information [file ADVS-9-2201104-s004.pdf]

Supporting Information

Cross-ray Ultrasound Tomography and Photoacoustic Tomography of Cerebral Hemodynamics in Rodents

*Shuai Na, Yang Zhang, and Lihong V. Wang**

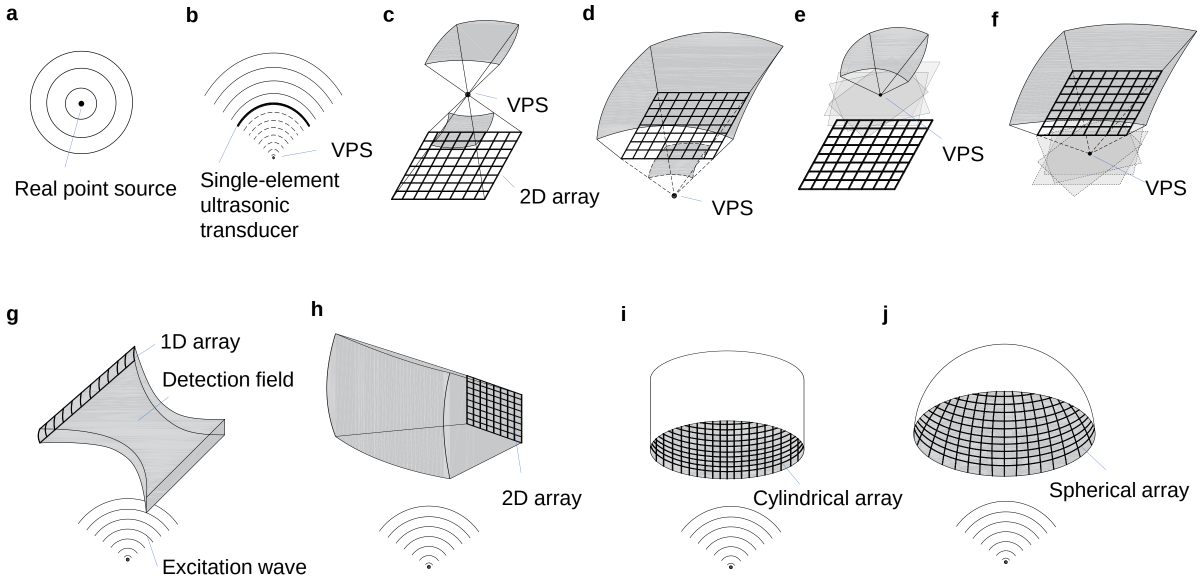

Figure S1. Possible transmission (top row) and detection (bottom row) configurations for CRUST. a, A real point source for transmission. **b**, A spherically focused single-element ultrasonic transducer with negative focusing for transmission. **c**, A 2D array with positive point focusing realized by digital beamforming. **d**, A 2D array with negative point focusing. **e**, A 2D array with plane-wave-synthesized positive point focusing. **f**, A 2D array with plane-wave-synthesized negative point focusing. Compared to those in (a) and (b), the configurations in (c–f) also allow for rapid digital scanning of the VPS. **g**, 2D imaging using a 1D linear array for detection. **h**, 3D imaging using a 2D array for detection. **i**, 3D imaging using a cylindrical array for detection. **j**, 3D imaging using a spherical array for detection.

Video S1. Conceptual demonstration of CRUST and conventional Doppler ultrasound imaging in vector flow imaging.

Video S2. CBF velocities of the mouse brain measured during hindlimb electrical stimulation with the intact scalp and skull. The top left panel shows the absolute CBF velocities over a stimulation cycle. The top middle panel shows the changes in the absolute blood flow velocities. The top right panel shows the changes in the blood flow velocity vectors. The bottom panel shows the average blood flow velocities in the blood vessels within the activated areas in Figure 9(f).

Video S3. CBF velocities of the mouse brain measured during hindlimb electrical stimulation with the scalp and skull removed. The top left panel shows the absolute CBF velocities over a stimulation cycle. The top middle panel shows the changes in the absolute blood flow velocities. The top right panel shows the changes in the blood flow velocity vectors. The bottom panel shows the average blood flow velocities in the blood vessels within the activated areas in Figure 10(f).
